# Usefulness and Limitations of Anti-S IgG Assay in Detecting Previous SARS-CoV-2 Breakthrough Infection in Fully Vaccinated Healthcare Workers

**DOI:** 10.3390/diagnostics12092152

**Published:** 2022-09-04

**Authors:** Gianluca Spiteri, Maria Grazia Lourdes Monaco, Gulser Caliskan, Angela Carta, Maria Diletta Pezzani, Giuseppe Lippi, Davide Gibellini, Giuseppe Verlato, Stefano Porru

**Affiliations:** 1 Occupational Medicine Unit, University Hospital of Verona, 37134 Verona, Italy; 2 Unit of Epidemiology and Medical Statistics, Department of Diagnostics and Public Health, University of Verona, 37134 Verona, Italy; 3 Section of Occupational Medicine, Department of Diagnostics and Public Health, University of Verona, 37134 Verona, Italy; 4 Infectious Diseases Unit, University Hospital of Verona, 37134 Verona, Italy; 5 Section of Clinical Biochemistry, Department of Neuroscience, Biomedicine and Movement, University of Verona, 37134 Verona, Italy; 6 Section of Microbiology, Department of Diagnostics and Public Health, University of Verona, 37129 Verona, Italy

**Keywords:** SARS-CoV-2 vaccination, SARS-CoV-2 breakthrough infection, COVID-19, anti-S IgG, healthcare workers

## Abstract

Introduction: The anti-spike (S) IgG assay is the most widely used method to assess the immunological response to COVID-19 vaccination. Several studies showed that subjects with perivaccination infection have higher anti-S IgG titers. However, a cut-off has not yet been identified so far for distinguishing infected subjects after vaccination. This study thus evaluates the performance of the anti-S IgG assay in identifying subjects with breakthrough infections (BIs) and its potential usefulness for screening healthcare workers (HCWs). Methods: Out of 6400 HCWs of the University Hospital of Verona vaccinated with two doses of BNT162b2, 4462 never infected before subjects who had completed primary vaccination were tested for IgG anti-S 6 to 9 months after the second dose. Of these, 59 (1.3%) had a BI. The discriminant power of IgG anti-S in detecting previous breakthrough infection was tested by constructing receiver operating characteristic (ROC) curves. Results: The discriminant power for BI was rather good (area under the curve (AUC), 0.78) and increased with decreasing time elapsed between antibody titer assessment and previous SARS-CoV-2 infection. Accuracy (AUC) sensitivity increased from 0.78 (95% CI 0.70–0.85) for BI in the previous six months to 0.83 (95% CI 0.67–0.99) for those in the previous two months, and from 0.68 to 0.80, respectively. The specificity (0.86) and optimal cut-off (935 BAU/mL) remained unchanged. However, BI were rather rare (1.3%), so the positive predictive value (PPV) was low. Only 40 of the 664 HCWs with antibody titer > 935 BAU/mL had previously confirmed BI, yielding a PPV of only 6.0%. When adopting as cut-off the 90th percentile (1180 BAU/mL), PPV increased to 7.9% (35/441). Conclusions: The anti-S IgG assay displayed good sensitivity and specificity in discriminating subjects with BI, especially in recent periods. However, BIs were rare among HCWs, so that the anti-S IgG assay may have low PPV in this setting, thus limiting the usefulness of this test as a screening tool for HCWs. Further studies are needed to identify more effective markers of a previous infection in vaccinated subjects.

## 1. Introduction

Since the beginning of the severe acute respiratory syndrome coronavirus 2 (SARS-CoV-2) pandemic, the scientific community has made huge efforts to develop effective vaccines. The first approved by the European Medicines Agency (EMA) was the mRNA-based BNT162b2 (Pfizer) vaccine [1]. Since 27 December 2020, the vaccination campaign across Europe has been rolled out throughout different priority groups, including healthcare workers (HCWs) [2,3]. As of 8 July 2022, over 49 million Italian citizens have received at least one dose of the coronavirus disease 2019 (COVID-19) vaccine (91.5% of the entire population), and almost 40 million also received a first booster dose, with the vast majority with mRNA vaccines [4]. Spike glycoprotein (S) is the major SARS-CoV-2 surface protein and the main player in viral penetration into the host cell. Its sequence was preferentially used to manufacture the most currently available vaccines, including BNT162b2 [5].

Several studies demonstrated that vaccination, even after the first dose, is effective in inducing a high humoral response except for a subset of high-risk populations (i.e., immunocompromised). A Greek study on 425 HCWs, of whom 63 (14.8%) were previously infected, evaluated the antibody titer for the receptor binding domain (RBD) of the S1 subunit 14 days after the administration of the first dose. A positive assay was reported in 92.2% of subjects, and higher levels were found in previously infected HCWs [6]. An Italian study involving 17,257 HCWs within the framework of the ORCHESTRA project, showed that a humoral response could be elicited in as many as 99.3% of all subjects 21–90 days after the first dose. The titer of previously infected subjects was positive in all cases [7]. Several factors, such as age, gender, previous infection before or after vaccination, and the number of doses, may influence anti-S IgG titers in vaccinated subjects. In particular, lower levels were found in elderly individuals, while subjects with previous diagnoses of SARS-CoV-2 infection had a higher titer [8,9]. The time passed after administration is another factor that impacts antibody levels. According to a literature review, the antibody titer peaks at 21–28 days after the second dose, decreasing to 55–85% of the peak value 140–160 days afterwards [10].

Previous breakthrough infection (BI) strongly affects the risk of reinfection. This information is essential to evaluate the recommendation for administering booster doses and estimating the real incidence and prevalence of SARS-CoV-2 infections throughout the different phases of the ongoing pandemic [11,12]. The gold standard for diagnosing SARS-CoV-2 infection is real-time quantitative polymerase chain reaction (RT-qPCR). However, the widespread use of this technique has some well-known drawbacks, such as costs, operator dependency, and sensitivity (in most cases, positivity is detectable only for a short time, typically between 10–15 days after symptoms onset). Furthermore, the sensitivity is even lower in asymptomatic infections, and its performance could also be impaired by mutations present in some SARS-CoV-2 variants (e.g., the so-called “S gene dropout”) [13].

Alternatively, the prevalence of previous SARS-CoV-2 infections can be assessed by detecting anti-SARS-CoV-2 antibodies. The mostly used serological test for this purpose involves the assessment of antibodies against the nucleocapsid protein (anti-N). However, this method has several limitations, and its reliability is still unclear. Demmer et al., in a study assessing the accuracy of a nucleocapsid-based assay, reported 100% sensitivity and 90% specificity in detecting recent SARS-CoV-2 infections [14]. An even higher specificity (100%) was found in a German study involving 80 vaccinated subjects [15].

Furthermore, Mizoue et al. reported that the sensitivity of anti-N antibodies was not related to symptoms [16]. On the other hand, Allen et al., in a study involving over 4000 HCWs, showed that, of the 23 subjects who had had BI shortly after the second dose (median 30 days), all had detectable anti-S antibodies. In contrast, only 6 (26%) had detectable anti-N antibodies, underlining their lower sensitivity. The assay based on anti-N antibodies also showed lower specificity than anti-S did, as the risk of cross-reactions was higher with protein N than with protein S [17]. Tutukina et al. measured IgG antibodies against N antigen and RBD in 47 subjects previously infected by SARS-CoV-2. All but one had positive values of anti-RBD, while only 34 were positive for the N antigen. In particular, the 26 subjects with no or mild symptoms were all positive for anti-RBD, but only half were positive for anti-N IgG. As a possible explanation, anti-S antibodies were hypothesized to be quickly released after SARS-CoV-2 infection, while anti-N antibodies are produced only after the intracellular viral invasion. This pattern is especially evident in mild forms of infection, where the viral replication is low, as it is the consequent release of N proteins [18]. This trend could be even stronger in vaccinated subjects since the early immunological response sustained by circulating antibodies further limits the entry of the virus into the host cells, the consequent production of N protein and the ensuing generation of anti-N antibodies.

Regarding the duration of circulating antibodies, the results are still inconclusive. Shrotri et al. reported that the anti-N IgG titer is stable in the short term (up to 3 months), but significantly drops in the medium-long term. Therefore, the anti-N titer seems to be more sensitive than the anti-S titer is in detecting early infection, but less sensitive post-recovery [19]. Accordingly, in a Dutch study, the median decay time after SARS-CoV-2 infection was two years for the anti-S titer, but less than one year for the anti-N titer. Accordingly, the rate of negative tests one year after nonsevere infections was negligible for anti-S antibodies but not for anti-N (3.4% versus 12.1%) [20]. An interesting study by Nakagama et al. evaluated serum antinucleocapsid antibody levels in 38 convalescent individuals 18 months after SARS-CoV-2 infection. The seropositivity rate at the end varied between 42% and 92%, depending on the type of assay used [21].

Anti-S antibodies could, therefore, be considered to be more reliable in identifying BI, especially in the medium and long term. Indeed, before BI, the anti-S titer does not show differences between subjects with or without BI; after infection, the titer was significantly higher in individuals with breakthrough infections than those without [22]. However, since antibodies are also produced following vaccination, it would be very useful to identify a cut-off value, enabling us to distinguish between vaccination responses with or without BI. Jabal et al. used an arbitrary threshold of 1000 AU/mL, and found that anti-S IgG titers above such value 6–8 months after completing primary vaccination were strongly suggestive of BI in the previous 3 months, displaying a positive predictive value of 93.3%. However, these findings were obtained on a relatively low sample of HCWs (n = 535), with a considerably high incidence of BI (around 20%) [23].

The present study aims at (i) verifying the potential usefulness of anti-S IgG assays as a screening tool for previous SARS-CoV-2 BI on a larger population of HCWs with a lower incidence of BIs (around 1%), (ii) identifying the optimal cut-off for detecting previous BI, in individuals SARS-CoV-2 naïve before vaccination, and (iii) verifying whether the diagnostic accuracy of anti-S IgG antibodies changes over time from vaccination or previous BI.

## 2. Materials and Methods

### 2.1. Setting, Population, and Testing

This study was conducted at the University Hospital of Verona, which employed 7638 HCWs in 2021. The study is also part of the ORCHESTRA project [24]. The present analysis was limited to 6404 HCWs who had voluntarily received two doses of the mRNA-based BNT162b2 vaccine. Of these, 615 and 20 HCWs were excluded as they were infected by SARS-CoV-2 before vaccination or after antibody assessment, respectively.

RT-qPCR performed diagnosis of infection. HCWs were tested regularly for periodic screening (every 10 or 20 days in high and low-risk wards, respectively), and following clinical suspicion and after strict contact with a positive case.

Of the remaining 5769 individuals, 4462 (77.3%) underwent a serological test to assess anti-S IgG titer from July to October 2021, after a median lag of 191 days (p25–p75 = 186–199 days) after the second dose. The humoral response was evaluated using the Liaison SARS-CoV-2 TrimericS IgG test (Diasorin), a chemiluminescence immunoassay (CLIA) for quantitatively detection of antitrimeric spike protein-specific IgG antibodies according to the manufacturer’s instructions. Test results were reported as BAU/mL (binding antibody unit per mL) after the 1:20 dilution of samples exceeding linearity range. The test was considered positive when the antibody level was ≥33.8 BAU/mL, as recommended by the manufacturer [25].

### 2.2. Statistical Analysis

The significance of differences between HCWs with or without BI was evaluated with Fisher’s exact test or χ2 test for categorical variables, and with the Wilcoxon–Mann–Whitney rank-sum test for continuous variables. The discriminant power of the IgG anti-S titer in detecting previous BI was tested by constructing receiver operating characteristic (ROC) curves and calculating the area under the curve (AUC). The optimal cut-off was chosen using the Liu method, which maximizes the product of sensitivity and specificity [26]. Calibration was accomplished by evaluating the risk of BI in different deciles of IgG anti-S titer. The analyses were repeated by considering BI in the 5, 4, 3, and 2 months preceding serum antibody assessment.

Multivariable analysis was performed using a logistic regression model, where BI was the response variable, anti-S IgG titer (coded as <90 or ≥90th percentile) the main predictor, and time elapsed since the administration of the two doses (<180 days, ≥180 days) as the main effect modifier, and sex, age, job title (physician, nurse, other HCW) as the potential confounders. The interaction between the anti-S IgG titer and the time elapsed since the administration of the second dose was also tested.

All analyses were performed using STATA^®^ version 17.0 (StataCorp, College Station, TX, USA).

### 2.3. Ethics

The research was performed following the 1964 Declaration of Helsinki standards and its later amendments. This research is part of the ORCHESTRA project that was approved (no. 436, 14 October 2021) by the Italian Medicine Agency (AIFA) and the Ethics Committee of the Italian National Institute of Infectious Diseases (INMI) Lazzaro Spallanzani. This research is also part of the SIEROPID study, approved by the Clinical Experimentation Ethics Committee of Verona and Rovigo (protocol no. 22851, 23 April 2020, and protocol no. 9594, 16 February 2021).

## 3. Results

The study population was aged 44.2 ± 11.9 years (mean ± SD; range 23–70 years) and consisted of 1228 men (27.5%) and 3234 women (72.5%). The majority of the study subjects were either nurses (n = 1617, 36.2%) or physicians (*n* = 1384, 31.0%), while other healthcare professionals (*n* = 675; 15.1%), technicians (*n* = 427; 9.6%), and administrative workers (*n* = 359; 8.1%) were less represented.

**Figure 1:**
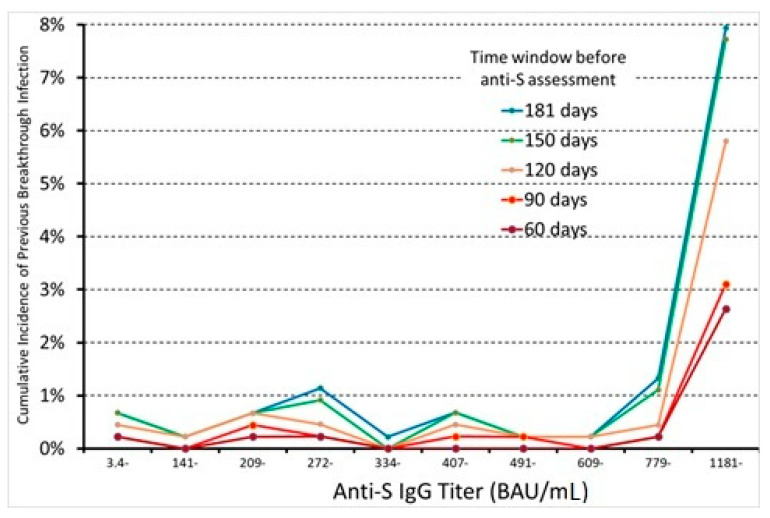
Cumulative incidence of previous BI as a function of anti-S IgG titer coded in deciles. Different curves were computed using different time windows for previous BI occurrences.

Of the HCWs, 59 (1.3%) were diagnosed with a BI. The probability of previous SARS-CoV-2 infection remained rather low (<1%) till the ninth decile of the anti-S IgG titer, increasing abruptly to 7.9% (35/441) in the last decile (1181–45600 BAU/mL) (Figure 1). The discriminant power for BI was fairly good (ROC-AUC = 0.78, 95% CI 0.70–0.85). At the best cut-off, sensitivity was 0.68, and specificity was 0.86 (Table 1).

When considering BI occurring in time windows of 150, 120, 90, and 60 days before anti-S antibody assessment, the number of cases decreased to 55 (1.23%), 39 (0.88%), 20 (0.45%), and 15 (0.34%), respectively, tending to concentrate in the upmost decile of anti-S IgG. Indeed, 40.3% of all BI cases (24/59) had a value of anti-S IgG below the 90th percentile, and this proportion decreased progressively to 38.2% (21/55), 35.9% (14/39), 35% (7/20), and 26.7% (4/15) when considering cases of BI infection occurring within 150, 120, 90, and 60 days before antibody assessment, respectively (Figure 1). The discriminant power of the anti-S titer thus increased inversely with the time window elapsed between the assessment of the antibody titer and the previous SARS-CoV-2 infection. Accordingly, ROC-AUC increased from 0.78 (0.70–0.85) for BI in the previous 6 months to 0.83 (95% CI 0.67–0.99) in the previous two months, and sensitivity from 0.68 to 0.80. On the other hand, specificity (0.86) and the optimal cut-off (935 BAU/mL) remained unvaried (Table 1).

The optimal cut-off slightly increased (up to 1275 BAU/mL) when considering the Youden index. However, this method, which maximises the sum of sensitivity and specificity, enabled higher specificity (0.86–0.92) at the expense of sensitivity, ranging from 0.64 to 0.80 (Table S1).

Notably, the positive predictive value (PPV) was remarkably low, as BIs were rather rare (1.3%). Of the 664 HCWs with antibody titer > 935 BAU/mL, only 40 had a previous confirmed BI, yielding a PPV of only 6.0%. When adopting the 90th percentile (1180 BAU/mL) as a cut-off, PPV increased to 7.9% (35/441). A simulation procedure showed that PPV would increase to 9.0%, 20.4%, 35.1%, 46.2%, and 54.8% with a cumulative incidence of 2%, 5%, 10%, 15%, and 20%, respectively, keeping sensitivity constant at 68% and specificity at 86%.

**Figure 2:**
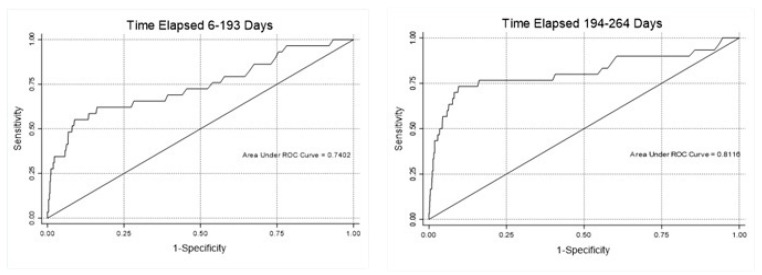
Discriminant power of antibody-S IgG titer evaluated by area under the ROC curve (ROC-AUC), as a function of time elapsed since second vaccination dose.

The discriminant power of the anti-S IgG titer increased with increasing elapsed time since the administration of the second dose. The ROC AUC was 0.74 (95% CI 0.62–0.85) when the elapsed time ranged from 6 to 193 days and increased to 0.81 (95% CI 0.70–0.91) thereafter (Table 2 and Figure 2).

**Figure 3:**
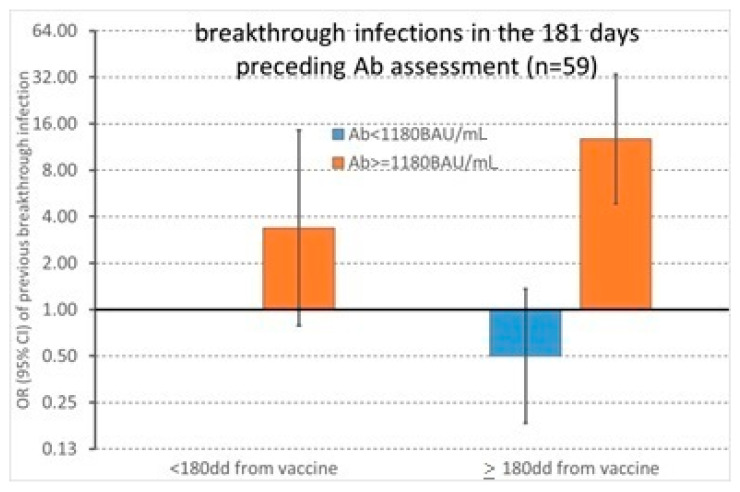
Odds ratios of previous breakthrough infection with corresponding 95% confidence intervals were computed with a logistic regression model controlling for sex, age, and job title.

These findings were confirmed in multivariable analysis, where the interaction between the anti-S IgG titer and elapsed time between the second vaccine dose and antibody assessment was significant (*p* = 0.011) (Figure 3). The OR of previous breakthrough infection was 3.38 (95% CI 0.79–14.51) in people with anti-S IgG titer greater than 1180 BAU/mL when the test was performed <180 days from vaccination, and 25.50 (13.89–46.82) when the test was performed thereafter.

## 4. Discussion

From the present study, we might infer a number of theoretically valuable conclusions. First, the probability of a previous BI did not seem to increase in parallel with a gradual increase in anti-S IgG titer, but rather it suddenly rose in the last decile above the threshold of 1180 BAU/mL. Then, anti-S IgG titer had good discriminant power for BI even in a low-incidence setting (around 1.3%), and its diagnostic usefulness may be further improved when baseline antibody titer is low, as in people who received the last vaccine dose more than six months ago, and when the immunological response elicited by BI is still sustained, as for BI occurring in the previous 2–3 months. The optimal cut-off for detecting previous BI ranges was in the range of 935–1275 BAU/mL according to the used statistical approach. However, the usefulness of anti-S IgG titer as a diagnostic tool for previous BI was relatively limited by the low PPV (6–8%) in a low SARS-CoV-2 incidence setting.

The immunological response elicited by COVID-19 vaccination tends to fade after a few months. As a consequence, the anti-S IgG titer generally decreases unless a BI occurs. This pattern fosters the opportunity to identify a cut-off for detecting a previous infection. Abu Jabal et al. identified an arbitrary 1000 AU/mL cut-off in their pioneering study [23]. The present study investigated this aspect and found an optimal cut-off of 935 or 1275 BAU/mL according to the statistical method used, and the anti-S IgG titer could better detect recent BI that occurred in the previous trimester than BIs found earlier.

The previous study published by Abu Jabal et al. demonstrated a good PPV (93%) of the anti-S IgG assay in a setting with a high incidence of BI (around 20%) [23]. By contrast, the very low incidence of BI in our hospital setting (i.e., 1.3%) determined a considerable reduction in PPV (about 8%), thus limiting the usefulness of anti-S IgG assessment as a screening tool for previous BIs in vaccinated HCWs. Hence, in low-risk populations, anti-SARS-CoV-2 S IgG antibody assessment should be used together with other parameters, namely, personal history and suggestive symptoms.

It could be hypothesized that a better approach to identify a previous BI could rely on a mixed strategy. Anti-N antibodies could be used in the first months after vaccination, as they are rather specific to natural infection, while anti-S titers are usually very high in response to vaccination irrespective of BI. Anti-S antibodies could be employed for identifying previous BI in the medium–long term (i.e., six months after vaccination), when anti-N titer usually declines. The same conclusion was reported by Dörschug et al., who suggested a combination of anti-spike protein- and antinucleocapsid-based serology as a useful option for discriminating between vaccination response and natural infection [15].

Furthermore, since the onset of this ongoing pandemic, several antibodies against viral proteins (along with anti-S and anti-N) have been investigated for improving the sensitivity and specificity of detecting previous infections. ORF8 and ORF3b antibodies proved to be effective in the disease’s initial stages. Furthermore, they displayed stability over time (at least until 100 days after the onset of symptoms). Long-term antibody persistence, as reported by the authors, is still under evaluation [27]. The study of Wang et al. also found out that the ORF8 protein was very immunogenic, displaying early seropositivity for IgM, IgG, and IgA. Particularly relevant was the presence of these antibodies in asymptomatic patients [28].

Some limitations should be acknowledged in the present study. First, the number of BIs was limited (i.e., 59), and this limited the statistical power of subgroup analyses. Moreover, anti-N titration was no longer available in our facility (dismissed because current indications endorse the only assessment of anti-spike antibodies for monitoring BNT162b2 reactivity), so we were unable to perform a side-by-side comparison of the two assays in our cohort. Lastly, we could not compare test accuracy and optimal thresholds in symptomatic and asymptomatic individuals due to the limited availability of clinical information. As symptomatic infections induce a larger and more persistent humoral response [8], a higher optimal anti-S IgG threshold could be expected. Test accuracy could hold even for previous BIs that are distant in time.

The present study has several strengths. The study population comprised nearly 4500 individuals who had completed the primary vaccination cycle, and had undergone a strict health surveillance program, which included swabs after close contact and/or symptom onset or at regular intervals. It is likely that most BIs could be detected, either symptomatic or not.

## 5. Conclusions

The anti-S IgG assay using a 935 BAU/mL cut-off showed good sensitivity and specificity in discriminating subjects with BI, especially in recent periods in fully vaccinated individuals for over six months. However, BIs were rare in the present setting, so the anti-S IgG assay had low PPV, limiting the usefulness of the anti-S IgG assay as a screening tool for HCWs. An improved approach combining anti-S/anti-N titers and regular swabs through appropriate statistical methods could be helpful in properly assessing BIs after the booster dose, which was followed by a high incidence of BIs in the Western world. Further studies are needed to identify more effective markers of previous infection in vaccinated subjects.

## Figures and Tables

**Table 1:** Discriminant power of antibody-S IgG titer to SARS-CoV-2 in predicting breakthrough infection, evaluated by area under the ROC curve (ROC-AUC). Discriminant power was computed for different time intervals preceding antibody assessment.

Best Cut-Off						
Elapsed Time (Days)	N	BI Cases	ROC (AUC)	Cut-Off Value	Se	Sp
All (13–181)	4462	59 (1.32%)	0.777	935	0.68	0.86
<150 (13–148)	4458	55 (1.23%)	0.785	938.5	0.69	0.86
120 days	4442	39 (0.88%)	0.784	935	0.69	0.86
90 days	4432	20 (0.45%)	0.795	935	0.70	0.86
60 days	4418	15 (0.34%)	0.831	935	0.80	0.86

Se = sensitivity; Sp = specificity.

**Table 2:** Discriminant power of antibody-S IgG titer, evaluated as a function of time elapsed since second vaccination dose.

Best Cut-Off					
Elapsed Time (Days)	N	ROC (AUC)	Cut-Off Value	Se	Sp
6–193	2629	0.7402	935	0.62	0.84
194–264	1832	0.8116	1035	0.73	0.82

Se = sensitivity; Sp = specificity.

## Data Availability

The datasets generated during the current study are not publicly available because they contain sensitive data to be treated under data protection laws and regulations. Appropriate forms of data sharing can be arranged after a reasonable request to the PI.

## Supplementary Materials

The following supporting information can be downloaded at: https://www.mdpi.com/article/10.3390/diagnostics12092152/s1. Figure S1: ROC curves to predict BI in time windows of 180, 150, 120, 90, 60 days before antibody assessment; Figure S2: sensitivity and specificity of anti-S IgG in predicting BIs in the 120 days preceding antibody assessment. Table S1: Optimal cut-off of anti-S titer to detect previous BI as a function of the method used and time window preceding antibody assessment; Table S2: Optimal cut-off of anti-S titer elapsed time since second vaccination dose as a function of the method used and time window preceding antibody assessment.
