# Supplementary material for: Usefulness and Limitations of Anti-S IgG Assay in Detecting Previous SARS-CoV-2 Breakthrough Infection in Fully Vaccinated Healthcare Workers
Source: Diagnostics (Basel). 2022 Sep 4;12(9):2152. doi: 10.3390/diagnostics12092152 (PMC9497628; doi:10.3390/diagnostics12092152)
Supplement: Supplementary file 1 [file diagnostics-12-02152-s001.zip › diagnostics-1877961-supplementary.pdf]

diagnostics

MDPI

Article

Usefulness and Limitations of Anti-S IgG Assay in Detecting Previous SARS-Cov-2 Breakthrough Infection in Fully Vaccinated Healthcare Workers

SUPPLEMENTARY MATERIAL

Table S1. Optimal cut-off of anti-S titer to detect previous BI, as a function of the method used and time window preceding antibody assessment.

Time window preceding ab assessment	Method	Empirical optimal cutpoint	Sensitivity at cutpoint	Specificity at cutpoint	Area under ROC curve at cut point
**180**	**Liu**	935	0.68	0.86	0.77
	**Youden**	1035	0.66	0.88	0.77
	**Near**	935	0.68	0.86	0.77
**150**	**Liu**	935	0.69	0.86	0.77
	**Youden**	1035	0.67	0.88	0.78
	**Near**	935	0.69	0.86	0.77
**120**	**Liu**	935	0.69	0.86	0.78
	**Youden**	1275	0.64	0.92	0.78
	**Near**	935	0.69	0.86	0.78
**90**	**Liu**	935	0.70	0.86	0.78
	**Youden**	1275	0.65	0.92	0.78
	**Near**	935	0.70	0.86	0.78
**60**	**Liu**	935	0.80	0.86	0.83
	**Youden**	935	0.80	0.86	0.83
	**Near**	935	0.80	0.86	0.83

Table S2. Optimal cut-off of anti-S titer time elapsed since vaccination 2nd dose, as a function of the method used and time window preceding antibody assessment.

Time window preceding ab assessment	Method	Empirical optimal cutpoint	Sensitivity at cutpoint	Specificity at cutpoint	Area under ROC curve at cut point
**6-193**	**Liu**	935	0.62	0.84	0.73
	**Youden**	1035	0.55	0.91	0.73
	**Near**	935	0.62	0.84	0.73
**194-264**	**Liu**	1035	0.73	0.90	0.82
	**Youden**	1035	0.73	0.90	0.82
	**Near**	1035	0.73	0.90	0.82

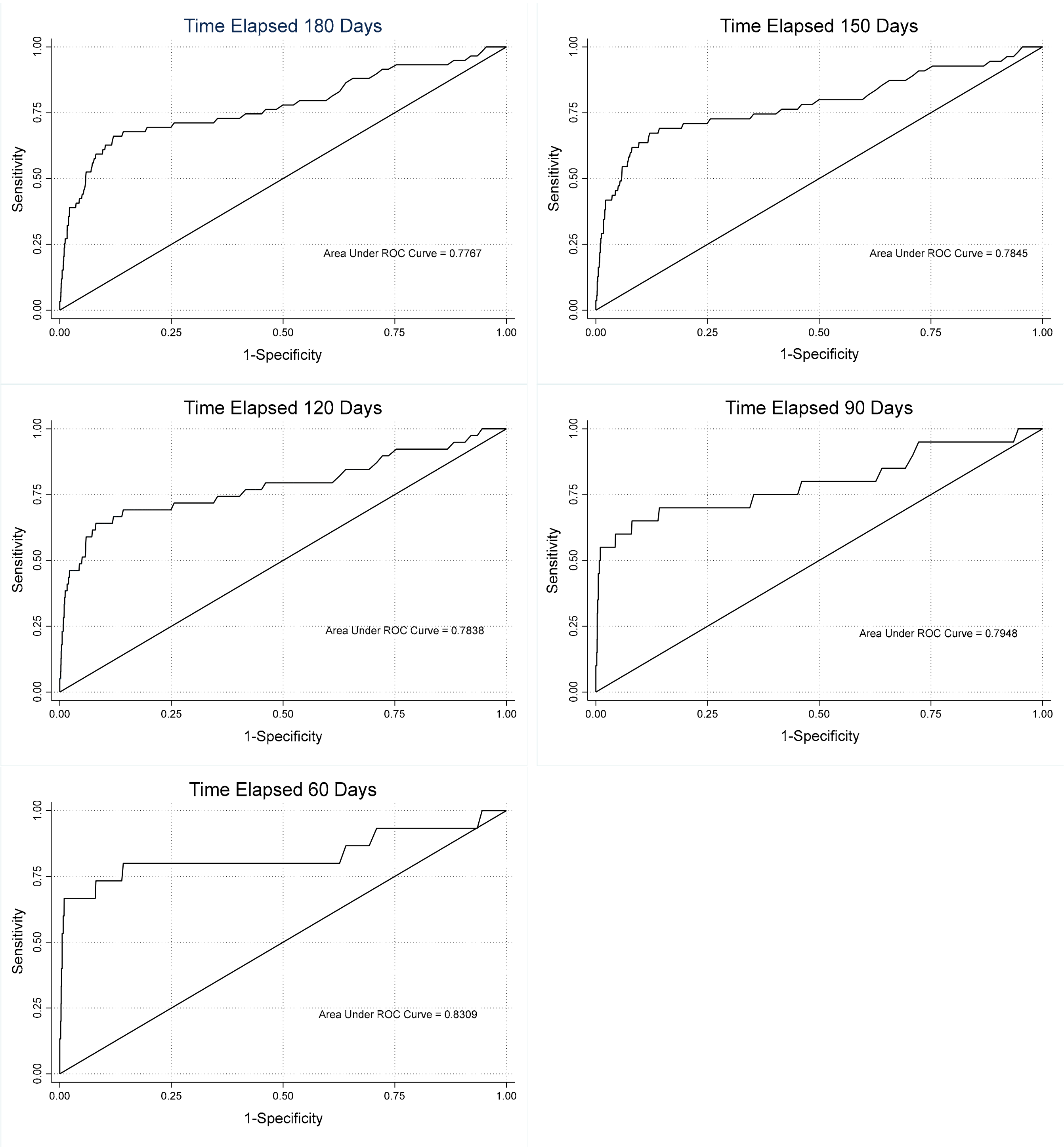

Figure S1. ROC curves to predict BI in time windows of 180, 150, 120, 90, 60 days before antibody assessment.

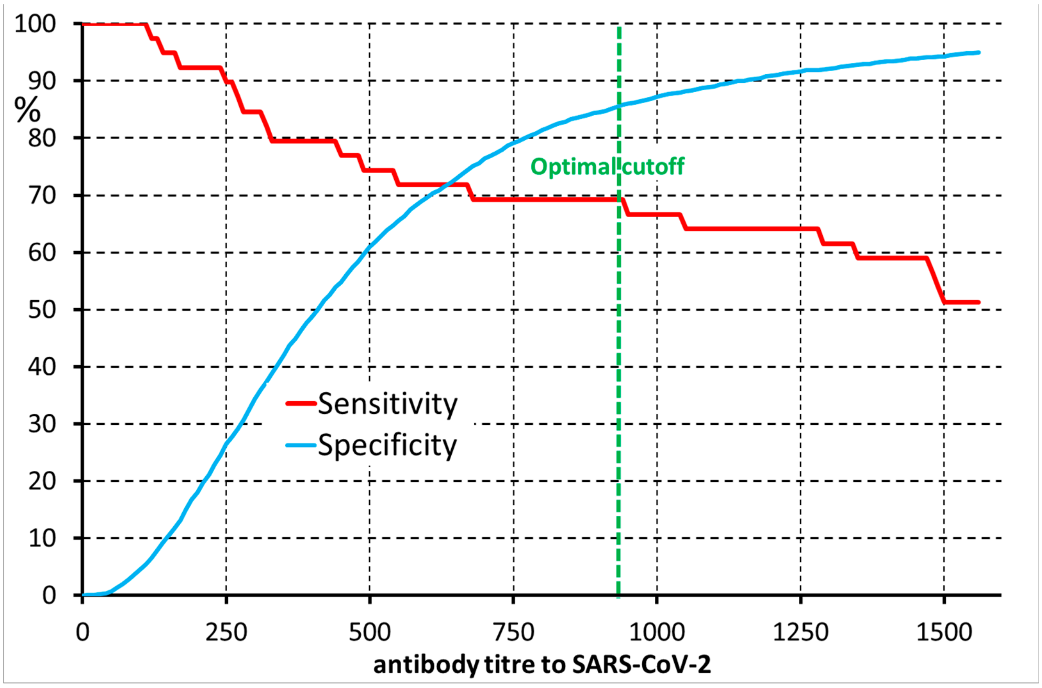

Figure S2. Sensitivity and specificity of anti-S IgG in predicting BI in the 120 days preceding antibody assessment.
